# miRNA-based biomarkers, therapies, and resistance in Cancer

**DOI:** 10.7150/ijbs.47203

**Published:** 2020-07-19

**Authors:** Boxue He, Zhenyu Zhao, Qidong Cai, Yuqian Zhang, Pengfei Zhang, Shuai Shi, Hui Xie, Xiong Peng, Wei Yin, Yongguang Tao, Xiang Wang

**Affiliations:** 1 Department of Thoracic Surgery, The Second Xiangya Hospital, Central South University, Changsha, Hunan 410011, China.; 2 Hunan Key Laboratory of Early Diagnosis and Precision Therapy, Department of Thoracic Surgery, The Second Xiangya Hospital, Central South University, Changsha, Hunan 410011, China.; 3 Key Laboratory of Carcinogenesis and Cancer Invasion, Ministry of Education, Department of Pathology, Xiangya Hospital, Central South University, Hunan, 410078 China.; 4 NHC Key Laboratory of Carcinogenesis (Central South University), Cancer Research Institute and School of Basic Medicine, Central South University, Changsha, Hunan, 410078 China.

**Keywords:** MicroRNAs, Cancer, miRNA-based biomarkers, miRNA-based therapies, Cancer therapy resistance

## Abstract

MicroRNAs (miRNAs), small non-coding RNAs (ncRNAs) of about 22 nucleotides in size, play important roles in gene regulation, and their dysregulation is implicated in human diseases including cancer. A variety of miRNAs could take roles in the cancer progression, participate in the process of tumor immune, and function with miRNA sponges. During the last two decades, the connection between miRNAs and various cancers has been widely researched. Based on evidence about miRNA, numerous potential cancer biomarkers for the diagnosis and prognosis have been put forward, providing a new perspective on cancer screening. Besides, there are several miRNA-based therapies among different cancers being conducted, advanced treatments such as the combination of synergistic strategies and the use of complementary miRNAs provide significant clinical benefits to cancer patients potentially. Furthermore, it is demonstrated that many miRNAs are engaged in the resistance of cancer therapies with their complex underlying regulatory mechanisms, whose comprehensive cognition can help clinicians and improve patient prognosis. With the belief that studies about miRNAs in human cancer would have great clinical implications, we attempt to summarize the current situation and potential development prospects in this review.

## Introduction

MicroRNAs (miRNAs) are small, evolutionarily conserved, non-coding RNAs (ncRNAs) of about 22 nucleotides in size that take significant roles in gene regulation 1. It was first described in 1993 that such ncRNAs were made in the proper timing of Caenorhabditis elegans 2. Later, many miRNAs were discovered in species such as animals and plants. There are about 40 thousand entries of hairpin forerunner forms in the 2018 version (miRBase 22), demonstrating about 50 thousand mature miRNAs in 271 species (http:www.mirbase.org) 3. It has been estimated that miRNAs can target more than 60% of human protein-coding genes 4. And nowadays, more than 2,000 miRNAs have been illustrated to have the ability to regulate the expression situation of about one-third genes in the human genome 5.

To be mentioned, the online miRNA resource miRBase provides systematic nomenclature for miRNAs dedicatedly 3. Take “hsa-miR-136-5p” for instance, “hsa” indicates the organism, “miR” signifies mature miRNA while precursors would be shown as “mir”, and “5p” there means this miRNA is from the 5' arm whereas “3p” would refer to those from the 3' arm when two miRNAs from a single predicted precursor present similar relative abundancies. Besides, a suffixed letter was used to distinguish close-related miRNAs, which we can see in miR-34a, miR-29b, miR-519c, among others. In keeping with miRBase, the HUGO Gene Nomenclature Committee (HGNC) also makes special gene symbols for miRNA genes 6, 7, which we have not introduced in detail here.

The control of gene expression with miRNAs is owned to virtually all known cancer cells. In the course of tumor development, miRNAs have shown its biology characteristics, indicating the possibilities in cancer classification and prognosis improvement. In this Review, we represent the biogenesis and regulation of miRNAs, as well as describe how their functioning mode works, with the links to cancer development. We also summarize the findings in which miRNAs act as biomarkers for cancer diagnosis and cancer prognosis prediction, and highlight the situation of miRNA-based cancer therapies.

## Biogenesis, regulation, and functioning mode of miRNA

### Biogenesis of miRNA

#### Canonical biogenesis

**Figure 1:**
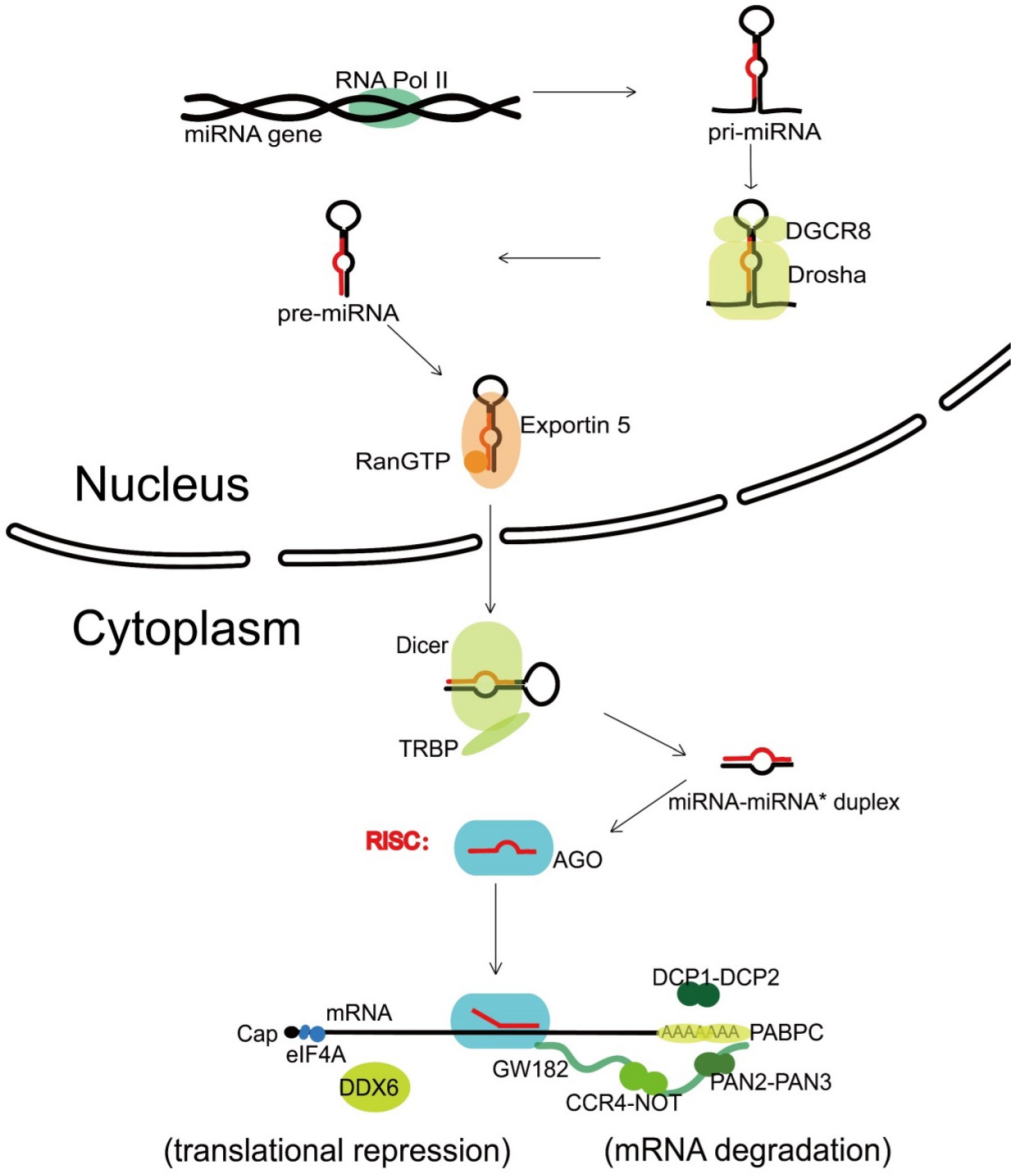
** Canonical miRNA biogenesis and miRNA-mediated gene silencing.** Transcribed by RNA Pol II, one nuclear miRNA gene produces a pri-miRNA, which is then recognized and cleaved into pre-miRNA by a microprocessor basically consisting of Drosha and DGCR8. Next, the pre-miRNA is exported to the cytoplasm with the help of Exportin 5 and RanGTP, further recognized and cut by Dicer and TRBP, and generates the miRNA-miRNA* duplex. After loaded into an Ago protein, the miRNA* strand is commonly degraded, while the miRNA strand forms the functional RISC. Upon loaded into RISC, the mature miRNA guides RISC to complementary target sequences located generally in the 3' untranslated region (3'-UTR) of mRNAs, then can function with the mode of mRNA degradation or translational repression. In the mRNA degradation mode, by the interactions with Ago protein, the GW182 recruits PABPC and binds the cytoplasmic deadenylase complexes PAN2-PAN3 and CCR4-NOT, rapid 5′-3′ mRNA degradation can also be in progress with the participation of DCP1-DCP2 complex. In the translational repression mode, the interaction of GW182 and CCR4-NOT can recruit DDX6 (a known translational inhibitor), and the release of eIF4AI or eIF4AII can inhibit the translation initiation. RNA Pol II, RNA polymerase II; DGCR8, DiGeorge syndrome critical region gene 8; RanGTP, Ran guanosine triphosphate; TRBP, HIV-1 TAR RNA binding protein; Ago, Argonaute; RISC, RNA-induced silencing complex; PABPC, polyadenylatebinding protein; PAN2-PAN3, poly(A)-nuclease deadenylation complex subunit 2 (PAN2)-PAN3; CCR4-NOT, carbon catabolite repressor protein 4 (CCR4)-NOT; DCP1-DCP2, mRNA-decapping enzyme subunit 1 (DCP1)-DCP2; DDX6, DEAD-box protein 6; eIF4A, eukaryotic translation initiation factor 4 A.

The canonical biogenesis of miRNA is a complex pathway with both nuclear and cytoplasmic steps. Transcribed by the RNA polymerase II (Pol II) 8, one nuclear miRNA gene produces a hairpin intermediate called “pri-miRNA” 9, which is then recognized by a microprocessor basically made up by one molecule of Drosha (a kind of RNase III enzyme with two RNase III domains) and two molecules of its cofactor, DiGeorge syndrome critical region gene 8 (DGCR8) 10. Next, Drosha cleaves two strands of the stem in the pri-miRNA hairpin, and form a stem-loop named “pre-miRNA” 11. Following that, the pre-miRNA formed in the nucleus is transported to the cytoplasm via Exportin 5 and Ran guanosine triphosphate (RanGTP) 12. In the cytoplasm, the pre-miRNA is further recognized by Dicer, an endonuclease with two RNase III domains too, and associates with a partner protein called TRBP (HIV-1 TAR RNA binding protein, also named as TARBP2) 13-15. Dicer cuts off the terminal loop from the hairpin and generates the miRNA-miRNA* duplex, in which the “miRNA*” is a passenger strand paired to the miRNA. The miRNA-miRNA* duplex is then loaded into an Argonaute (Ago) family protein and is unwound driven by the N-terminal domain of Ago 16. Notably, in the process of assorting among Ago complexes, the nucleotide composition of miRNA-miRNA* duplex also impacts on the strand selection 17. Finally, the miRNA* strand is commonly degraded, while the other strand, also known as guide strand (to become the mature form of miRNA) is retained and form the functional RNA-induced silencing complex (RISC) eventually *(Figure 1).*

#### Non-canonical biogenesis

The re-evaluation about the roles of key factors in the canonical biogenesis shows that there are alternative mechanisms for miRNA biogenesis 18. In fact, there are more and more miRNAs with non-canonical biogenesis reported as more high-throughput sequencing datasets are used, and those miRNAs characterized as Drosha-independent or Dicer-independent.

Drosha-independent miRNAs were classified into three classes. The first is “mirtrons”, which refer to certain debranched introns that feature as pre-miRNAs and can be exported out of the nucleus immediately for Dicer-mediated processing 19. Evidence showed mammalian mirtrons have many features different from Drosha-generated pre-miRNAs, such as substantially longer mirtron hairpins, frequently 3'-uridylation, and atypically heterogeneous 5' termini 20. The mirtron pathway is a permanent class of non-canonical biogenesis and is thought to provide an option for the miRNA emergence prior to the advent of Drosha 19. A second class is originated from other ncRNAs like small nucleolar RNAs (snoRNAs) and transfer RNAs (tRNAs) 21, 22, whose gene transcription often relies on the transcriptional and processing key factors of various RNAs to help produce the hairpins serving as Dicer substrates 1. However, although some snoRNA and tRNA fragments can originate potential miRNAs, and even be loaded into RISC, most Ago-associated snoRNA and tRNA fragments may remain other functions unidentified now 23. For example, studies suggested the DNA damage response (DDR) could be mediated by such ncRNAs 24, 25. Besides, a kind of tRNA-derived fragments was demonstrated to enhance mRNA translation by regulating ribosomal proteins such as RPS28 26, 27. The third class involves 7-methylguanosine (m7G)-capped pre-miRNAs, which are synthesized from the 5' end of genes by Pol II directly, characterized by small RNA Cap-Seq, and exported by Exportin 1 28.

Dicer-independent miRNAs are much fewer compared to Drosha-independent ones. Such miRNA biogenesis method needs Ago2 slicer catalytic activity 29. A typical small RNA taking a great role in erythropoiesis, miR-451, is processed by Drosha but does not require Dicer for maturation. Its pre-miRNA gets cleaved by the catalytic center of the Ago loaded, generating an intermediate 3' end which is further trimmed by poly(A)-specific ribonuclease (PARN) 30, 31.

#### Regulation of microRNA biogenesis

As described in the canonical pathway, efficient miRNAs biogenesis relies on a series of elements and proper local RNA structures. In particular, the RISC slicer Ago2 can cut the pre-miRNA to additional processing intermediate termed ac-pre-miRNA, which can work as a Dicer substrate to mature into the active miRNA, which may facilitate nicked passenger strand removal 32. Regulatory post-translational modifications (PTMs), like the phosphorylation, ubiquitylation, and sumoylation of miRNA biogenesis factors, can also affect miRNA processing by connecting miRNA expression with cell signaling pathways 23. For example, a newly published study indicated that through the PTM of DDX17 (a cofactor of the Drosha-DGCR8 complex), miRNA biogenesis and histone modifications can get concerted regulation, which underlies many cancer stem-like features 33. The target messenger RNA (mRNA) can also regulate the biogenesis of cognate miRNAs 34. Besides, several recent studies suggested that RNA-​binding proteins (RBPs) and long non-coding RNAs (lncRNAs) can control miRNAs biogenesis 35, 36. Even, a lncRNA of hyper-conservative regional origin was illustrated to block pri-miRNA cleavage by Drosha 37. In brief, the biogenesis process of miRNAs is in tight temporal and spatial regulation, and faulty biogenesis in human body would be associated with a great number of diseases, particularly cancer.

### Effects of miRNA

RISC is an effector ribonucleoprotein complex, in which the miRNA works as a target-recognition element, an Ago family protein serves as a vital component, and a range of accessory components are along with them 38. RISC assembly is a key process for miRNAs exerting functions, in which four Ago proteins named Ago1-4 encoded by the mammalian genome are available. Ago2 particularly, with additional special features introduced ahead, is the most highly expressed. Human miRNAs are thought to associate with all Ago proteins, even though some of them preferentially loaded into specific Ago proteins 39.

Generally, miRNAs are guided to specific target sites which are located in the 3'-untranslated region (3'-UTR) of mRNAs, then direct their post-transcriptional repression effects with the mode of mRNA degradation or translational repression. Nucleotide 1 of miRNA counted from 5' end is permanently unavailable for pairing, while complement miRNA nucleotides 2-8 form the seed which is pivotal for the recognition of target mRNA 40. In the target mRNA, an adenine (A) opposite to miRNA nucleotide 1 (known as “t1A”) is recognized by an Ago binding pocket and confers enhanced target repression, supplementing the seed 41. Although target sites that complement miRNA nucleotides 2-8 with t1A are the strongest, those with complementarity to miRNA nucleotides 2-7 or 3-8 are considered canonical too, as non-canonical sites (which lack a contiguous 6 nucleotides pairing to the seed region) are thought inefficacy 42. By the way, in a subset of sites, the 3′ end pairing can make help for target recognition, particularly nucleotides 13-16 (the supplementary region) 43. Recent findings have also shown that 3′ end pairing on uridylated miRNAs can also contribute to novel target recognition 44.

The mRNA decay requires the consecutive action of a 182 kDa glycine-tryptophan protein (GW182) and the mRNA-decapping enzyme subunit 1 (DCP1)-DCP2 complex 45. Following the multivalent protein actions with Ago proteins 46, GW182 recruits cytosolic polyadenylate-binding protein (PABPC), which promotes mRNA deadenylation through binding the poly(A)-nuclease deadenylation complex subunit 2 (PAN2)-PAN3 complex and carbon catabolite repressor protein 4 (CCR4)-NOT complex 47. Deadenylation is followed by decapping via the DCP1-DCP2 complex, leading to rapid mRNA degradation with 5′-3′ exoribonuclease 1 (XRN1) 48. Apart from the degradation of miRNA targets, GW182-mediated interaction with CCR4-NOT can also promote translational repression by the recruitment of a known translational inhibitor, the DEAD-box ATPase DEAD-box protein 6 (DDX6) 49. And the translation initiation can be inhibited by releasing eukaryotic translation initiation factor 4 A-I (eIF4AI, the founding member of DEAD-box helicases) or its paralogue eIF4AII from target mRNAs 50, which is proved to be GW182 independent in Drosophila even 51. However, in 2018, a study reported novel miRNA-recognition elements (MREs) that only work in protein coding sequences (CDS) regions, which is in requirement of extensive 3' side base-pairing rather than the 5' seed, and is Ago-dependent but GW182-independent 52; In 2016, another study reported that Ago Tryptophan-binding pockets mediating GW182 binding are required for translation inhibition in human cells as well as D. melanogaster cells 53, both showing the complete mechanism of translational repression still remains unresolved *(Figure 1)*.

It is revealed that two modes of miRNA-mediated gene silencing are connected with each other 54, and global steady-state measurements verified that the effect of mRNA decay dominates for generally explaining 66% - >90% of such silencing 55. Besides, there are complex interaction networks formed by miRNAs, as each miRNA can influence widespread genes with the advantage of their small size, and multiple miRNAs can silence the same gene, although the effect of each miRNA on each gene is relatively mild 43. Typically, the miRNA abundance in any cell is less than the effective target abundance (the count of sites that have to be combined when achieving half-maximal de-inhibition on the targets), which illustrates why lowly expressed miRNAs function negligibly and suggests that even the most repressed genes can be silenced by increased miRNAs 1. By reinforcing normal transcripts and attenuating abnormal transcriptional programs, miRNAs can buffer against random fluctuations in the post-transcriptional level 56. What needs to be emphasized is, cytoplasmic miRNAs can localize in the endoplasmic reticulum, mitochondria, and other compartments with some differences in their pathways 57.

Intriguingly, different from cytoplasmic functions mentioned above, miRNA can affect regulation both post-transcriptionally and transcriptionally in nuclear by working with promoters in three models including RNA-RNA hybrid, RNA-DNA hybrid, and RNA-DNA triplex 58. Besides, nucleus miRNAs can serve as enhancer triggers to activate gene transcription epigenetically 59. All of those are related to Ago proteins. Recently, a study illustrated that overexpressed nuclear Ago in embryonic stem cells has an important place at the beginning of differentiation 60. Furthermore, nucleoplasmic protein Sfpq can directly modulate nucleoplasmic and cytoplasmic miRNA functions 61. These findings present a complicated miRNA-related modulation network, and it will be more perfect as new discoveries continuing to appear.

Cell-free miRNAs found in various biological fluids indicate that miRNAs could contribute to intercellular signaling, and are potential biomarkers available for a variety of diseases 62. Exosomes, the naturally equipped biological vehicles, have been thought to contain most circulating miRNAs but with some controversy 39, and adipose tissue is proved to constitutes a primary source for circulating exosomal miRNAs 63. Besides, although exosomal Ago2-loaded miRNAs and single-stranded miRNAs have been detected, evidence of cell-cell communication via exosomal miRNAs in a physiological context remains elusive 39. Recently, a study found miR-494 was up-expressed in exosomes of melanoma samples, which can be reduced by Rab27a depletion 64, showing the feasibility of transferred exosome-shuttled miRNAs as therapeutic methods. Thus, we require more studies to fully understand their mechanism and function.

## Biological functions of miRNA in cancer

### miRNAs in cancer progression

In 2002, the deletion and low-expression of miR-15 and miR-16 cluster in chronic lymphocytic leukemia were demonstrated, firstly suggesting the role of miRNAs in the progression of cancers 65. During the past period, miRNAs have been linked to virtually all known cancer processes. According to the target gene, some miRNAs often negatively influence protein-coding oncogenes, while some other miRNAs can inhibit known tumor suppressors, therefore miRNAs could act as onco-miRNAs or tumor-suppressor miRNAs 66.

The dysregulation of miRNAs can also link to alterations in genes that govern cancer progression. During the formation and progression of gastric cancer, the expression of miR-1269 is upregulated, which promotes cancer cell proliferation as well as cell cycle G1-S transition and suppresses cell apoptosis by regulation of the AKT signaling pathway and the Bax/Bcl-2 signaling pathway, with the target of RASSF9 67. In oral squamous cell carcinoma (OSCC) patients, miR-9 is downregulated, whose expression induces G0/G1 cell cycle arrest, while the use of miR-9 mimics significantly halts cell proliferation by repressing cyclin-dependent kinase 6 (CDK6) and cyclin D1 68. Besides, miR-9 has an expanded scope of target RNAs because of its pri-miRNA paralogs 69. As one of the most abundant miRNAs in the brain, miR-9 was verified to target COL18A1, THBS2, PTCH1, and PHD3 directly in glioma cells, whose overexpression takes positive roles in processes such as proliferation and cell cycle progression 70. Therefore, the regulatory characterization of miRNAs in the cell cycle regulation is complex, maybe with some critical pathways still to be found.

As for cell proliferation, it was found that the miR-145 targeting A disintegrin and metalloproteinase 17 (ADAM17) could inhibit such a process in liver cancer cells 71. Another study about hepatocellular carcinoma (HCC) showed overexpressed miR-487a happened in poor prognostic patients, which increases cell proliferation by phosphoinositide-3-Kinase regulatory subunit 1 (PIK3R1) induced AKT signaling 72. Those findings can provide novel promising treatment strategies in the therapy of HCC, as well as other cancers.

Several miRNAs can take roles in programmed cell death, especially in the apoptosis of cancer cells, too. The dysregulation of p53 has been widely reported to enable cancer cells against apoptosis, which could be triggered by the low-expression of miR-192, miR-194, and miR-215 in multiple myeloma 73. It was also shown the p53 diversification happened because of several miRNAs in a research of soft tissue sarcoma as well as breast cancer 74, while some Epstein-Barr virus (EBV) miRNAs were associated with p53 regulation in nasopharyngeal cancer as well as EBV-associated gastric cancer 75. Besides, miR-205 and miR-338-3p can restrain the apoptosis of prostate cancer cells by targeting one of its inhibition genes, B-cell lymphoma 2 (BCL-2) 76. In the extrinsic apoptotic pathway, miRNAs can inhibit pivotal elements like Fas ligand (FasL). It was characterized that FasL is directly targeted by miR-21-5p, and the expression of its mRNA and protein both can be negatively influenced by the up-regulation of miR-21-5p in HCC cells 77.

**Figure 2:**
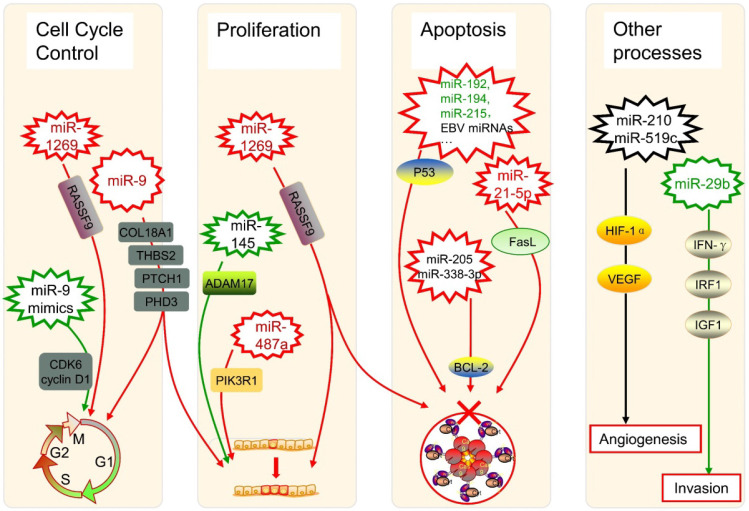
** Several miRNAs in cancer progression.** The up-expression or down-expression of miRNAs (in red or green, if mentioned) could influence specific cancer progression process (red frames and lines mean promote cancer progression, while green ones mean inhibit). RASSF9: Ras-association domain family 9; CDK6: cyclin-dependent kinase 6; EBV: Epstein-Barr virus; BCL-2: B-cell lymphoma 2; FasL: Fas ligand; ADAM17: A disintegrin and metalloproteinase 17; PIK3R1: phosphoinositide-3-Kinase regulatory subunit 1; HIF-1α: Hypoxia-inducible factor 1α; VEGF: vascular endothelial growth factor; IFN-γ: interferon-γ; IRF1: interferon regulatory factor 1; IGF1: insulin-like growth factor 1.

The angiogenesis of tumors can be controlled by miRNAs such as miR-210 and miR-519c under hypoxic conditions via modulating Hypoxia-inducible factor 1α (HIF-1α) and vascular endothelial growth factor (VEGF) 78. Regulatory networks of miRNAs are considered to be a characteristic of epithelial-mesenchymal transition (EMT) in the physiopathology of OSCC 79. An aggressive phenotype of colorectal cancer (CRC) is significantly associated with depression of miR-29b, whose existence restricts the development and incursion of cancer cells, with the potential interaction of interferon-γ (IFN-γ), IRF1, and IGF1 80. All those highlighted showed that miRNAs are implicated in tumor progression including cell cycle, cell proliferation, cell apoptosis, angiogenesis, EMT, and tumor invasion, acting important and complex roles in the regulatory network of cancers *(Figure 2)*.

### miRNAs affect tumor immunity

The dysregulation of patient immune systems is significant in cancer development, and a variety of miRNAs implicate in the process of tumor immune surveillance as well as tumor immune escape. As discussed ahead, miRNAs can be loaded by exosomes and then involved in intercellular communication. In cancer, exosomes can affect many aspects including the progression, metastasis, multidrug resistance, and EMT 81. Some kinds of immune cells can be directly interacted with, such as the function of natural killer (NK) cells limited by miR-23a, the function of dendritic cells diminished by miR-212-3p and miR-203, and the inhibition of CD8^+^ cells while the activation of CD4^+^ regulatory T cells effected by tumor-derived miR-24-3p 81. Especially, talking about NK cells, emerging evidence shows that some miRNAs can, directly and indirectly, modulate their anti-tumor potential for instance by controlling the surface expression of immune checkpoints on NK cells or that of their ligands on tumor cells 82-85.

Apart from the oncogenic and tumor-suppressive function, miRNAs were reported to impress on tumor immunogenicity and antitumor immune responses. For example, a study confirmed that miR-124-3p is the target of LINC00240, and could promote cervical cancer development with the action of STAT3 and MHC class I-related proteins A (MICA), mediating natural killer T (NKT) cell tolerance 86. While, under the HLA class I antigen processing machinery (APM) components, regulation of the B7 family members, and expression of IFN-γ signaling molecules, other miRNAs including miR-346, miR-451, and miR-148a are summarized to be implicated 81. In fact, miRNAs are interacted with many immune checkpoint proteins (ICPs) other than the B7 family 87, presenting significant advantages for the personalized treatment and prognostic prediction of cancer patients.

What is more, miRNAs have become the critical regulators for antitumor immune cells. Here we present several related studies. For tumor-infiltrating lymphocytes (TILs), scientists characterized the tight connection among miR-574-3p, PD-L1 expression, and TIL levels in chordoma 88, while mechanically another group has presented the function of the p53/miR-34/PD-L1 axis in immune evasion of lung cancer 89. For myeloid-derived suppressor cells (MDSCs), miR-34a can also regulate oncoprotein MUC1 and then results in the MDSC increase in acute myeloid leukemia (AML) 90. For tumor-associated macrophages (TAMs), miR-98 directly targets the gene of interleukin 10 (IL-10) in TAMs, suppressing the progress of HCC 91. For cancer-associated fibroblasts (CAFs), miR-21 acts a role in the metabolically altering process of CAFs thereby affects pancreatic cancer cells 92.

### Involved miRNA sponges in cancer

A pool of different transcripts that compete synergistically to attract miRNAs for interactions is known as competing endogenous RNAs (ceRNAs), mainly including some experimentally confirmed ncRNAs, even some pseudogenes and protein-coding RNAs. Through the titration mechanism, the ceRNAs can regulate each other indirectly, while ceRNA-ceRNA interactions are also called miRNA sponge interactions 93.

In addition to miRNAs, ncRNAs contain several types including lncRNAs, circular RNA (circRNAs), small nuclear RNAs (snRNAs), and so on, making up 98% in the human genome 94. Among these, lncRNA is the one first found as miRNA sponge 93, and many recent studies illustrated the role lncRNAs take in miRNA-related tumor regulation. In a study about lung adenocarcinoma stem cells, miR-146 is referred to as the post-transcriptional regulation of NUMB, while one of its sponge TUSC-7, a strong suppressive lncRNA, could abolish the degradation toward to NUMB by inactivating NOTCH signaling. This research proved the decrease of tumor-suppressive miR-146 was essential in TUSC-7 function, showing complex non-coding genes employed in renewal repression regulation 95. In nasopharyngeal carcinoma (NPC), the lncRNA FAM225A was over-expressed as a sponge of miR-590-3p and miR-1275, promoting NPC development by up-regulating *integrin β3*, the target of the two miRNAs 96. Besides, a novel lncRNA, prostate cancer-associated transcript 7 (PCAT7), was considered to promote the tumor progression of NPC as a miR-134-5p sponge 97. In addition to these, the miRNA sponge-lncRNAs have also been studied in numerous kinds of cancers such as a miR-34a sponge MALAT1 in melanoma 98, a miR-15a/16 sponge LINC00461 in multiple myeloma 99, a miR-330-5p sponge LINC00958 in pancreatic cancer 100, a miR-7-5p sponge RP4 in colorectal cancer 101, a miR-324-5p sponge TPT1-AS1 102 and a miR-186 sponge antisense noncoding RNA of the INK4 locus (ANRIL) 103 in cervical cancer, a miR-96 sponge TP53TG1 in pancreatic ductal adenocarcinoma 104, and a miR-300 sponge TUG1 in gallbladder carcinoma (GBC) 105.

circRNAs are some endogenous ncRNAs with a covalently closed successive circle, working as miRNA sponges is one of its important roles 106. The circRNAs expressions were abnormal in tumor cell lines, tissues, and even plasma samples of cancer patients with the advantage of RNA sequencing technology 107. In one study, circMMP9 was found to act as a sponge directly targeting miR-124, quickening up the growth and migration of glioblastoma multiforme (GBM) cells. Furthermore, they found that the cyclization and increased expression of circMMP9 were induced by eukaryotic initiation factor 4A3 (eIF4A3), whereas eIF4A3 tied up with the mRNA transcript of MMP9 in GBM 108. Besides, circRNAs were verified as miRNA sponges in OSCC 109, HCC 110, lung cancer 111, breast cancer 112, gastric cancer 113, and so on.

Pseudogenes are genomic elements that lose protein-coding abilities because of various endogenous inactivating mutations 114 and some of them have also been found to be miRNA sponges such as PTENP1 and OCT4P4 115. Some types of protein-coding RNAs can sponge to miRNAs, too 93. The tumor suppressor gene PTEN has been demonstrated to be a miRNA sponge 116, while TNRC6B was found to be a PTEN ceRNA then, showing hints at complex interactions among different pathways in cancer 117. Apart from them, an antisense RNA of the TP73 gene, TP73-AS1, was verified to sponge to human-specific miRNA miR-941 by Ago protein immunoprecipitation experiments, wound-healing assay, and massive transcriptome data analyses. Moreover, the duo represents of TP73-AS1 and miR-941 showed a fast alteration of noncoding regulators that influences cell migration, proliferation, and tumorigenesis 118.

However, some questions had been raised about the hypothesis of ceRNAs. In 2014, a stoichiometry on miR-122 and its targets shown little influence exerted by ceRNAs 119. Another study on global properties also doubted the sponge function of the vast majority of circRNAs 120. Furthermore, researchers found the phenomenon of target mRNAs or ncRNAs triggering miRNA tailing, trimming, and decay, which was termed as target RNA-directed miRNA degradation (TDMD) 121. A recent study showed this is a process triggered by target RNAs with an enzymatic attack activity from an Ago2 PAZ domain around miRNA 3' end 122. Compare to ceRNAs, TDMD needs much fewer target RNAs to make obvious miRNA reduction 123. Based on the TDMD study, a regulatory network containing a lncRNA (Cyrano), a circRNA (Cdr1as), and two miRNAs (miR-7, miR-671) was even established for neuronal activity 124. In short, we think further investigations are needed to reach a consensus and theorem about those ncRNAs participating in the biological action of miRNAs in cancer.

## Clinical utilization of miRNA

### Potential miRNA-based biomarkers

As so many functions miRNAs have in cancer development, the dysregulation of one specific miRNA or a group of miRNAs may be closely associated with human cancer progression. There are thousands of papers published by scientists all over the world in PubMed about potential miRNAs that are available for cancer diagnosis and prognosis. We searched recent journal articles reported since Jan 1st, 2017, and presented some of them here (*Table 1*). What should be noted is, a lot of them were not verified by Northern Blot—the gold standard for analyzing RNA, so the utilization of those potential biomarkers in clinical practice at once is not suitable. However, these studies might provide novel insights and promising tools for us.

#### miRNA-based biomarkers for diagnosis

The miRNAs from different sample sources might have idiographic meanings. The urinary cell-free miRNAs are appropriate potential markers for some urologic neoplasms, for example, researchers performed a multicenter study by 543 urine samples and proposed a noninvasive diagnostic tool with a > 90% (*p* < 0.001) sensitivity to discriminate bladder cancer from benign hematuria, which could reduce unnecessary cystoscopies at a certain point 125. Another study even reported the relationship between urinary levels of miR-126 and children proliferating hemangiomas 126. Besides, a research of head and neck squamous cell carcinoma (HNSCC) put forward a combination of seven methylations of genomic loci encoding microRNA (mgmiRs) with sensitivity and specificity of 76.7% and 86.1% respectively in saliva, even though lower than that in tissues as 92.6% and 92.4%, still a novel and promising screening tool 127. What is interesting, the measurement of several miRNAs on cerebrospinal fluid (CSF) samples suggests high sensitivity as well as specificity for the distinction of primary central nervous system lymphoma (PCNSL) 128. And, some doctors came up with a feasible idea of detecting significant miRNAs on fine-needle aspiration (FNA) samples with the method of endoscopic ultrasonography for pancreatic cancer early detection 129. These sources like urine, saliva, CSF, FNA samples, as well as common serum or plasma used for miRNA detection showed us excellent potential in their clinical applications.

A variety of detection methods were used to explore the potential miRNA biomarkers. What impressed us most is the electrochemical nano-genosensor. Such nano-genosensors had a remarkable signal amplification effect, resulting from the unique structure of nitrogen-doped functionalized graphene, nano-sized silver particles, and polyaniline 130. By applying differential pulse voltammetry (DPV), researchers performed a detection after the functionalizing of that nanocomposite and immobilizing of the circumstantial miR-21 aminated complementary oligonucleotide sequence, as miR-21 is a foregone biomarker in breast cancer. By virtue of highly sensitive and label-free, that nano-biosensor is utilizable for breast cancer early discovery via detecting the miR-21 on human samples directly without the need for the pretreatment, RNA extraction, or amplification 130. Equally exciting is, some researchers exploited a digital amplification-free quantification manner by the use of NanoString Technologies, the nCounter technology, which can identify dysregulated exosomal serum miRNAs (ex-miRNAs) for progressive prostate cancer 131. The above studies provide new ideas, and we look forward to more advanced technologies applied to the detection of miRNAs.

Those diagnosis methods put out based on miRNAs, though with some differences, have great significance. Unlike those who use sole miRNA as biomarkers, some scientists identified a panel of miRNAs for a kind of cancer, like the six miRNAs significantly overexpressed in gastric cancer 132. Three exosomal miRNAs together also presented higher accuracy for the diagnosis of metastatic lung cancer than a single one 133. After the verification of Northern Blot and TaqMan miRNA qRT-PCR assays, scientists noticed the considerably higher ratio of miR-25/92a group to the miR-22/29a group in cervical specimens with cervical intraepithelial neoplasia, then proposed an early diagnostic method for cervical cancers 134. Another group identified the DNA methylation of genomic loci encoding miRNAs (mgmiRs) *in vitro* cell lines, and successfully developed a seven-mgmiR diagnostic panel for HNSCC 127. A more instance, four miRNAs (miR-498, miR-183, miR-205, and miR-31), which are suitable indicators in urine for the presurgical identification of representative benign renal oncocytoma, can well conduct preoperative plans and avoid some unnecessary stress for patients 135. These miRNA panels may be more effective than sole miRNA used, but, both with outstanding significance for cancer patients.

However, there still exist challenges unresolved for the application of miRNAs as cancer diagnosis biomarkers. There are three plasma miRNAs (miR-222-3p, miR-423-5p, and miR-150-5p) for the detection of OSCC identified, former two of which are considered inversely associated with cancer progression related to T stage, lymph node metastasis, and clinical stage, while the latter one in plasma is up-regulated in OSCC. Nevertheless, what the Cancer Genome Atlas (TCGA) analysis reveals is not matched to their findings, as OSCC miR-222-3p increased in tissue whereas not in plasma, and over-expressed miR-150-5p just happened in plasma 136. Talking about the inconsistency of miRNA levels between plasma and tissue, the explain maybe the selective release of miRNA 137, or maybe various parameters (such as drugs) affecting circulating miRNAs 138, or maybe something we haven't discovered yet. Furthermore, as we introduced, there are many miRNA sponges involved in cancer so some of them may be used as biomarkers such as lncRNA MIR31HG (a miR-575 sponge) for HCC 139 and circRNA cTFRC (a ceRNA for miR-107) for bladder cancer 140, providing a new perspective on cancer diagnosis.

#### miRNA-based biomarkers for prognosis

Many articles concerning miRNA biomarkers have revealed the mechanisms behind. Publishing in an article about ESCC in April 2019, researchers first determined the miRNA and mRNA expression situations in ESCC tissues by the application of microarray; following this, they constructed a miRNA-mRNA regulatory network by multistep bioinformatics methods; then they measured miR-1 expression using *in situ* hybridization (ISH) and explored its connection with clinicopathological characteristics and outcome of ESCC patients. Moreover, they studied the functional role of miR-1 with several experiments and verified a direct target gene—Fibronectin 1 (FN1)—by applying technologies like qRT-PCR, Western blot, and luciferase reporter assays 141. In short, these results provide a valuable therapeutic target more than only a potential prognostic predictor.

As we mentioned earlier, miRNA sponges might be significant components for the post-transcriptional gene regulation, and some miRNA sponges have been indicated to be prospective biomarkers of cancer. For example, markedly up-regulated lncRNAs CCAT1—a sponge of let-7 miRNA family—is linked with AFP, tumor size, and microvascular invasion in HCC, which presented as a poor prognostic biomarker for HCC patients 142. With the consideration of different molecular profiles in breast cancer subtypes, researchers found the subtype-specific sponge interactions are quite distinct 143, which show high prognostic potential for breast cancer subtypes and may expand research perspectives for other tumors. By the way, another group analyzed the interactions of the lncRNA-mRNA ceRNA network based on the four subtypes of breast cancer systematically 144, providing new insights for other scientists.

Some researchers identified prognostic factors by using expressed databases such as TCGA and the Molecular Taxonomy of Breast Cancer International Consortium (METABRIC). For instance, the METABRIC cohort data was utilized to further explore the expression and survival data of miR-196a in breast cancer, following with genomic data analysis and chromatin interaction analysis 145. Not only can these databases save a lot of time and effort, but they also remove the need for patient consent or ethics approval.

Currently, computer-aided biomarker discovery is full of attractiveness. Take a novel network vulnerability analysis model reported for example, scientists identified five miRNAs as potential biological markers for prostate cancer metastasis based upon their bioinformatics model, and get convinced by the prediction action, reported articles, and functional enrichment analysis 146. As experimental methods are difficult to find the driver or pivotal molecules in the viewpoint of systems, such a concentrated network in the model can serve as a biological system for biomarker prediction, which focuses on special regulatory patterns of stability altering 146.

Taken together, as more and more miRNA biomarker candidates in cancers have been come up with, studies with larger cohorts are needed, and complete mechanisms of miRNA dysregulation are warranted to be understood.

### miRNA-based therapies

**Figure 3:**
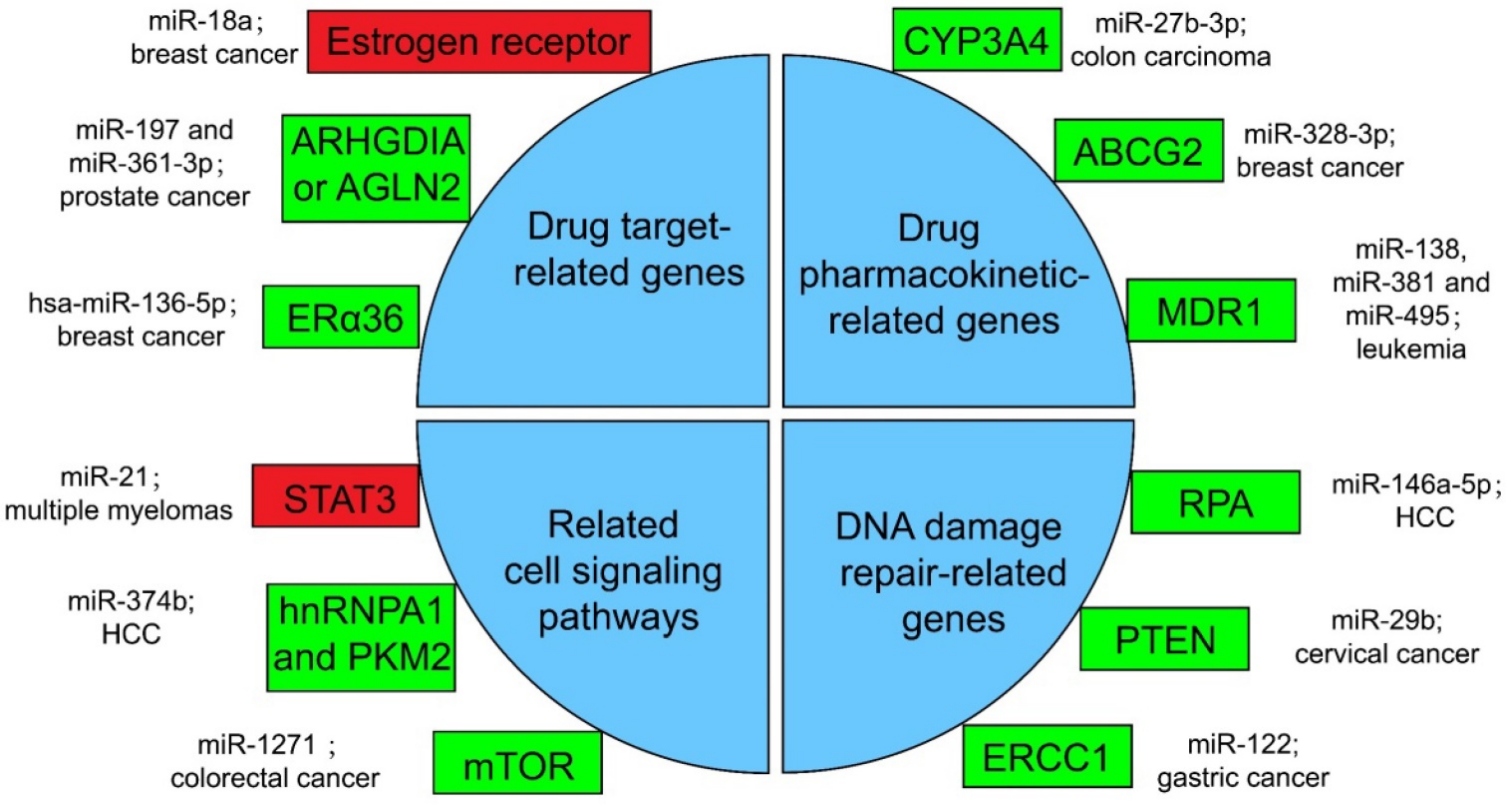
** Several miRNA-related cancer therapy resistance.** There are four categories along with some regulators presented in the text, where the rectangle in green or in red shows whether the function of miRNAs in mentioned cancer type is positive or negative for the corresponding therapy. ERα36: estrogen receptor alpha 36; CYP: cytochrome P450; ABCG2: ATP-binding cassette sub-family G member 2; MDR1: multidrug resistance 1; STAT3: signal transducer and transcriptional activating factor 3; MYC: myelocytomatosis oncogene; HnRNPA1: heterogeneous nuclear ribonucleoprotein A1; PKM2: the Pyruvate kinase muscle 2; mTOR: mammalian target of rapamycin; RPA: replication Protein A; PTEN: phosphatase and tensin homologous protein; ERCC1: excision repair cross-complementing 1.

As we discussed earlier, miRNAs play a non-negligible role in the regulation of cancers and can, therefore, be used as alternative therapeutic targets. Many miRNAs have been entered the clinical stage of cancer treatment, along with many related experiments *in vitro* and *in vivo*, as introduced later *(Table 2)*. Here, we would like to picture some advanced treatment methods including the following four forms *(Figure 3)*.

#### Combination of miRNA therapeutics and chemotherapy

Recently, numerous researches have been conducted on chemo with miRNA therapeutics. In completed pharmacological experiments, miR-126 inhibits CDK4/6 and PIK3CA to prevent cell cycle progression along with the mitosis. In related experiments, it has been confirmed that transfection of miR-126 mimic into breast cancer cells can enhance its sensitivity to fourteen chemotherapy drugs such as trimetinib and alpelisib, and prevent the emergence of drug resistance 147. Moreover, temozolomide-resistant glioblastoma cells sensitized by miR-151a was restored with the moderation of XRCC4-mediated DNA repair after the transfection of miR-151a mimic into the glioblastoma cell line 148. Investigators studied the tumor-suppressive function of miR-205 in gemcitabine-resistant pancreatic ductal adenocarcinoma, finding that lentiviral vectors can inhibit cancer stem cell proliferation and resensitize those gemcitabine-resistant cells when miR-205 is overexpressed. Thus, it supplied a platform for the combined treatment of miR-205 and gemcitabine as a feasible method to treat advanced pancreatic cancer 149. Another science group used pancreatic cancer miR-1291 prodrug with gemcitabine and nab-paclitaxel in cancer model mice, noticing that miR-1291 up-regulated ARID3B and further induced programmed cell death, DNA damage, and mitotic block, which significantly inhibited the growth of tumor cells 150. Correspondingly, it was confirmed that forced expression of miR-634 can induce programmed cell death in a variety of cell lines containing pancreatic cancer cells, and intravenous injection of lipid nanoparticles containing miR-634 can greatly reduce xenograft tumor progress in mouse BxPC-3 cells 151. In another study, raised miR-149 level restrained cell proliferation and colony formation in neuroblastoma cells, nevertheless promoted apoptosis and chemical sensitivity to doxorubicin (Dox) 152. Dox was also found to induce autophagy and interact with miR-137, while miR-137 can make it easier for pancreatic cancer cells to react with chemotherapy 153. The combinatorial strategy of anti-tumor miRNAs with chemo drugs can synergistically enhance the therapeutic efficacy, thus showing a hopeful research direction for cancer therapies.

#### Combination of miRNA therapeutics and radiotherapy

The prime mechanism of radiotherapy is inducing DNA double-strand break (DSB). It is known that miR-34a acts as a regulator in various human neoplasms 154. In an animal experiment, scientists studied the cooperative relationship between miR-34a and radiation therapy, finding that overexpression of miR-34a (which bound to the 3'-UTR site of RAD51) enhanced γ-H2AX foci and inhibited the formation of HR and RAD51 foci, thereby inhibiting tumor cell DNA repair. They transfected MRX34 (a liposomal nanoparticle bond to miR-34a mimics) into a mouse model of non-small cell lung cancer (NSCLC), leading to significantly enhanced radiotherapy effect and suppressed tumor cell growth 155.

The sharp phosphorylation of histone H2AX to γ-H2AX presents a biological marker for DNA repair after radiotherapy. miR-328-3p can directly interact with the 3'-UTR of H2AX transcript and reduce its protein expression level, whose overexpression promotes radiation-induced cellular DNA damage. Investigators then introduced miR-328-3p mimic into animal models of NSCLC, and the tumor cells were significantly reduced in mice after radiotherapy, proving that overexpressed miR-328-3p can improve cell radiosensitivity with the change of DNA damage/repair signaling pathways 156. In conclusion, miRNA targeting approaches are able to promote the sensitivity and effect of radiation therapy, and employing them together would potentially help many carcinoma patients improve their prognosis.

#### Combination of miRNA therapeutics and immunotherapy

Immunotherapy is one of the main treatments for current cancer treatments, one of its principles is to inject genetically modified tumor-specific T cells for malignant tumor patients to identify, aim, and kill tumor cells. However, the persistence and effectiveness of T cell therapy are not satisfactory. In addition, miRNAs are key controllers for T cell stimulation, proliferation, living, differentiation, and effector function, all of which are dominant causes for judgment of adoptive immunotherapy treatment. With the in-depth understanding of miRNA and the exploration of its application in treatment, the application of miRNA will solve many problems in immunotherapy 157. For example, miR-143 overexpression enhances specific killing of HER2-CAR (human epidermal growth factor receptor 2-chimeric antigen receptor) T cells which target esophageal carcinoma cell line TE-7, because miR-143 enhances the tumor-treat effect of T cells by supporting memory T cell differentiation and metabolic reprogramming via targeting glucose transporter 1 (Glut-1) gene 158. A novel lentiviral vector of one miRNA cluster and epidermal growth factor receptor (EGFR)-variant III was introduced into CAR T cells to enhance the durability of its therapeutic response in glioblastoma, which effectively improved the efficacy of adoptive T cells 159. Besides, it was illustrated that miR-1258 targeted the 3′-UTR of programmed death ligand 1 (PDL1), and thus affected the treatment of myeloma by inhibiting PDL1 160. With the introduction of miRNAs, we may deal with those difficulties in immunotherapies, and promote their application in human cancers.

#### Replacement therapy or miRNA mimics

Through research on miRNA-based cancer gene therapy, it has been found that single miRNA has limited therapeutic effects on cancer. This is because many miRNAs participate in the appearance and development of malignant neoplasms by regulating broad target genes or various signal transduction processes, and cancer cells can get tolerance to miRNA involvement within a special period and restore proliferative capability via other routes readily 161. Therefore, interventions targeting no less than two miRNAs with complementary relations may restrain multiform signaling pathways and improve the efficacy of miRNA mimic therapy.

Up-regulated miR-34a in cell line HCT116 remarkably restrained cell growth, migration, aggression, and metastasis, along with evoked apoptosis, G1 phase arrest, and p53 transcriptional state 162. Scientists have also effectively co-expressed miR-126 and miR-34a through oncolytic adenovirus vectors in a mouse model of pancreatic adenocarcinoma (PAC) and noticed that their co-expression can lead to enhanced or synergistic treatment functions in PAC 161.

Besides, up-regulated miR-34a can inhibit NSCLC regeneration and metastasis 163. Simultaneous addition of miR-34 and let-7 in a mouse model of NSCLC leads to extensive suppression of key oncogenes and brings survival advantages 164. MRX34, a miRNA mimic of miR-34a, is in phase I and phase II clinical trials for melanoma at present 165, as well as a phase I clinical trial for NSCLC 155. In another study, overexpression of E2F1 in high-grade tumors resulted in decreased outcome and chemoresistance of patients. Compared with the effect of transfection with miR-205-5p or miR-342-3p into tumor cell lines alone, the co-transfection of these two miRNAs into tumor cell lines inhibited E2F1 significantly more, thereby reducing the chemically resistant better 166, 167.

In summary, miRNA-based cancer therapies have shown promising results. Methods by combining with different strategies synergistically, applying complementary miRNAs together, or developing novel forms of miRNA provide significant clinical benefits to cancer patients potentially. Further investigations are needed in this field to achieve the ultimate goal of improving patient OS and DFS.

### miRNA-related cancer therapy resistance

It is well established that drug-resistance takes major responsibility for the failure of advanced cancer patients treated with chemotherapy. At present, the main drug resistance mechanism of cancer cells is the miRNA-targeted regulation of therapy-related mRNA 168. Specifically, it can work through the following four methods.

#### Resistance with drug target-related genes

Drugs against cancers are connected with target proteins, and abnormal regulation of these proteins often leads to drug resistance 168. It was confirmed that up-regulated miR-18a serves as a potential marker for bad overall survival (OS) and disease-free survival (DFS) after neoadjuvant chemotherapy (nCT), as miR-18a increased significantly in the remaining breast cancer cells after nCT. While researches proved that the remaining breast cancer cells are resistant to tamoxifen, which may associate the function of miR-18a to inhibit the translation of the estrogen receptor, but does not affect its transcription process. Moreover, miR-18a has been verified to link with the mitotic hallmarks and cell cycle checkpoints, and does not affect tumor invasion and migration 169. In addition, in the study of castration treatment of prostate cancer, investigators justified the increase of miR-197 and miR-361-3p could silence tumor suppressors ARHGDIA or TAGLN2. ARHGDIA or TAGLN2 can stabilize the activity of the androgen receptor, the level of mRNA and protein, and cause the effect of castrate-resistant. It was revealed that miR-197 and miR-361-3p can work as biological markers for castrate-resistant 170. Low level of hsa-miR-136-5p in tamoxifen treatment of breast cancer often indicates poor prognosis and metastasis of tumor cells. The expression of estrogen receptor alpha 36 (ERα36) was negatively related to the content of hsa-miR-136-5p. It has been found that the up-regulation of ERα36 is in connection with the inhibition of methylation in the promoter region, which may suggest that hypermethylation may inhibit miRNA biogenesis and processing, but the specific mechanism has not been clearly studied 171, 172.

Drug resistance according to the drug target-related genes regulated by miRNAs is the most direct reason for the failure of chemo agents. Only by excavating these regulators, can we then take actions such as choosing undisturbed drug targets or applying combined therapies.

#### Resistance with drug pharmacokinetic-related genes

Pharmacokinetics is the study facing the absorption, distribution, metabolism, or excretion (ADME) of drugs in the body 173. CYP3A4 is an important drugmetabolizing enzyme in the liver and intestine, which belongs to a group of well-known drug-metabolizing enzymes, cytochrome P450 (CYP) 174. CYP3A4 is often highly expressed in drug-resistant tumor samples, and the inhibition of CYP3A4 activity is a considerable part for solving the drug resistance of tumor cells 175. By the way, the content of miR-27b-3p was proved to be limited in drug-resistant tumor cells, while it has been proved that the up-regulation of miR-27b-3p can inhibit the expression of CYP3A4 and change the drug resistance of LS-180 cells—a kind of colon carcinoma cell line 176. Similarly, the expression level of miR-328-3p was low in breast cancer, but the ATP-binding cassette sub-family G member 2 (ABCG2) level was high. ABCG2 is a very important protein related to ADME. When it is highly expressed, it will accelerate the metabolism of mitoxantrone and produce drug resistance in tumor cells 177. When cancer cells overexpress miR-328-3p, it can obviously limit the expression of ABCG2 and decrease the drug resistance of breast cancer cells 176. Also, miR-27b can directly act on CYP1B1 to reduce its expression 178. miR-148a-3p, miR-148b-3p, or miR-152-3p can act on SPIN1 and inhibit its expression, then affect the drug metabolism enzyme CYP2C8, UGT2B4, UGT2B17, and ABCB4, and decrease the drug resistance in cancer cells 179.

As for the regulation of drug transport, overexpressed products of drug efflux pump genes take a vital part in drug resistance. Proteins such as ABCC1, ABCG2, ABCB1, and MRP1 can exclude drugs from tumor cells and keep tumor cells alive 180. miRNA can also directly or indirectly inhibit the overexpression of drug efflux pump genes, and decrease drug resistance. For example, for the treatment of leukemia patients, researchers found that miR-138 can restrain the expression of NF-kB/p65 and then inhibit the production of Multidrug resistance 1 (MDR1). MDR1 is a drug efflux pump P-glycoprotein (P-gp) that encodes proteins that lead to chemotherapy resistance in patients with leukemia. Therefore, overexpression of miR-138 can inhibit chemotherapy resistance in patients with leukemia 181. Likewise, miR-381 and miR-495 are low-expressed in drug-resistant cells of leukemia patients, too. Up-regulation of miR-381 and miR-495 can effectively inhibit the production of MDR1 and P-gp, and decrease the drug resistance of tumor cells in patients with leukemia 182.

The study of pharmacokinetics can help deal with the contradiction between the safety and efficacy of anti-cancer drugs because we must consider the appropriate dose for cancer patients. So it is significant to take miRNA regulations into consideration of ADME.

#### Resistance with related cell signaling pathways

A great number of anticancer drugs play a role by the inhibition of cell proliferation and the guidance of apoptosis. These mechanisms are inhibited to a certain extent in drug-resistant cells, resulting in drug resistance 168. Some miRNAs can influence drug resistance by controlling significant genes in cancer cell-related signaling pathways. For example, the signal transducer and transcriptional activating factor 3 (STAT3) functions importantly in multiple myelomas. The regulation of miR-21 is closely related to STAT3, which can specifically link with the enhancer part of miR-21 and enhance the expression of miR-21, whereas such miRNA is an anti-apoptotic factor and eventually leads to drug resistance in tumor cells 183. In the treatment of HCC, miR-374b can inhibit the expression of PKM2 by inhibiting the antagonistic glycolysis pathway, thus making resistant cells re-sensitive to sorafenib 184. HnRNPA1 and PKM2 were both highly expressed in sorafenib resistant cells 185. Among them, hnRNPA1 can promote the high expression of PKM2. Related studies have demonstrated the expression of miR-374b in drug-resistant HCC cells with a negative correlation with hnRNPA1 and PKM2. Down-regulation of hnRNPA1 *in vitro* can inhibit the expression of PKM2, but the mechanism has not been evidently studied 186. In a CRC study, the expression of mammalian target of rapamycin (mTOR) was increased in the remaining tumor cells after cisplatin (a non-specific cell cycle drug) treatment, while mTOR has been shown to participate in the progress and metastatic process of several cancers, behaving like a carcinogenic gene through a variety of mechanisms 187. In contrast, miR-1271 in tumor cells is low in CRC. And the overexpression of miR-1271 *in vitro* cell experiments will significantly inhibit the expression of mTOR, then decrease the drug resistance of tumor cells 188.

There are rather complicated regulation networks in multiple cancer cell development processes such as proliferation, metastasis, and apoptosis. We must realize that cancer treatment is an organic whole, and interventions that target only one aspect of the signaling pathway without considering related regulatory mechanisms may not achieve satisfactory results.

#### Resistance with DNA damage repair-related genes

In many drug-resistant cells, drugs achieve therapeutic effects by destroying tumor cell genes. However, some enzymes can repair these damaged DNAs, and produce drug resistance in tumor cells 175. In HCC cells, miR-146a-5p was usually expressed low, while Replication Protein A (RPA) was highly expressed 189. RPA can inhibit DNA repair pathway 190, and then promote tumor cell growth and resist apoptosis in radiotherapy of liver cancer. After radiotherapy, miR-146a-5p expression was raised in HCC cells, while RPA expression was suppressed, and the DNA repair pathway was activated to promote the sensitivity to radiotherapy 191. Similarly, the decrease of miR-29b level and the increase of phosphatase and tensin homologous protein (PTEN) expression in residual tumor cells happened after radiotherapy for cervical cancer. High expression of PTEN will inhibit the AKT signaling pathway, which will lead to DNA DSB repair, therefore produce tumor cells radioresistance 192. If miR-29b was overexpressed in the remaining cervical cancer cells after radiotherapy, it will significantly inhibit the expression of PTEN, activate the AKT signal pathway and inhibit DSB, and enhance the sensitivity to radiotherapy 193, 194. Besides, the miR-122 level was decreased in drug-resistant gastric cancer cells treated with cisplatin. On the other hand, miR-122 directly acted on excision repair cross-complementing 1 (ERCC1) 3′-UTR and inhibited its expression. Therefore, in drug-resistant tumor cells, the expression of ERCC1 will be increased, which can repair the genes of tumor cells destroyed during chemotherapy and produce drug resistance of tumor cells 195.

A comprehensive cognition of the roles miRNAs take in tumor resistance will help us improve existing therapies and explore new drugs, so that related researches can help clinicians and truly improve patient prognosis.

## Conclusion

The biogenesis of miRNA is an intricate activity classified into canonical and non-canonical pathway, many factors could influence the expression of miRNAs and their functions. Dysregulation of miRNAs can link to cancer development processes such as interfering with the cell cycle and evading immune destruction. Because of the ability of modulating tens to hundreds of specific genes, miRNAs as vital biomarkers in cancer diagnosis or prognosis prediction are appearing. Some kinds of miRNA-related cancer therapy resistance exist according to the control of drug target-related genes, drug pharmacokinetic-related genes, DNA damage repair-related genes, and related cell signaling pathways. Besides, miRNA therapeutics can be combined with chemotherapy, radiation therapy, and immunotherapy, providing new methods for miRNA-based therapy, as well as the use of replacement therapy and miRNA mimics. It is obvious that miRNAs, along with other ncRNAs such as lncRNAs and circRNAs, would have great clinical implications in the pathogenesis, diagnosis, and treatment of human cancers. More studies related should be followed to understand the mechanism completely and improve targeted therapies in cancer.

## Figures and Tables

**Table 1:** Potential miRNA biomarkers for diagnosis and prognosis in cancers

Cancer type	miRNA	Dysregulation	Sample type	Detection method	Ref.
**for diagnosis**					
Pheochromocytomas or paragangliomas (PPGLs)	miR-210	down	serum	qRT-PCR	196
Colorectal cancer (CRC)	miR-103a-3p, miR-127-3p, miR-151a-5p, miR-17-5p, miR-181a-5p, miR-18a-5p and miR-18b-5p	up	plasma	qRT-PCR	197
Cervical cancer	the ratio between miR-25/92a and miR-22/29a groups	up	cervical specimens	Northern Blot and TaqMan miR qRT-PCR assays	134
Gastric cancer	miR-491-5p	down	Tissue and serum	qRT-PCR	198
Invasive breast cancer	exosomal miR-223-3p	up	plasma exosomes	TaqMan miR assays	199
Glioma	miR-21, miR-222 and miR-124-3p	up	serum exosomes	qRT-PCR	200
breast cancer	miRNA-21	up	blood	electrochemical nano-genosensor	130
Lung cancer	miR-33a-5p and miR-128-3p	down	tissue and blood	qRT-PCR	201
Prostate cancer	miR-214	down	cell lines	qRT-PCR	202
Hepatocellular carcinoma (HCC)	miR-221	up	cell lines, tissues, and serum	qRT-PCR	203
Oral squamous cell carcinoma (OSCC)	miR-222-3p, and miR-423-5p / miR-150-5p	down/up	plasma	qRT-PCR	136
Head and neck squamous cell carcinoma (HNSCC)	mgmiR9-1, mgmiR124-1, mgmiR124-2, mgmiR124-3, mgmiR129-2, mgmiR137, and mgmiR148a	up	tissues, cell lines, and saliva	qMS-PCR	127
Primary central nervous system lymphoma (PCNSL)	miR-21, miR-19b, and miR-92a	up	cerebrospinal fluid (CSF)	RT-qPCR	128
Germ cell tumours (GCTs)	miR-371a-3p	up	serum	RT-qPCR	204
Synovial sarcoma (SS)	miR-92b-3p	up	serum and cell lines	miRNA microarray and RT-qPCR	205
**for poor prognosis**					
Esophageal squamous cell carcinoma (ESCC)	miR-1	down	tissues	ISH	141
Advanced cervical cancer	miR-944	up	cell lines and FFPE tissues	RT-qPCR	206
Oral squamous cell carcinoma (OSCC)	miR-1290	down	plasma	qRT-PCR	207
Colorectal cancer (CRC)	Circ_0026344 (a miRNA sponge for miR-21 and miR-31)	down	tissues and cell lines	qRT-PCR	208
Pancreatic ductal adenocarcinoma (PDAC)	exosomal miR-451a	up	plasma	TaqMan MiR assays	209
Small intestinal neuroendocrine tumors	miR-375	down	biopsies	ISH	210
TNM stage II and III colon cancer	a 16-miRNA signature including miR-143-5p, miR-27a-3p, miR-31-5p, miR-181a-5p, miR-30b-5p, miR-30d-5p, miR-146a-5p, miR-23a-3p, miR-150-5p, miR-210-3p, miR-25-3p, miR-196a-5p, miR-148a-3p, miR-222-3p, miR-30c-5p and miR-223-3p	up	fresh frozen biopsies	RT-qPCR	211
Gastric cancer	miR-155	up	tissues	qRT-PCR	212
Pancreatic ductal adenocarcinoma (PDAC)	miR-296-5p	up	tissues and cell lines	miRNA array and RT-PCR	213
Ovarian cancer	miR-135a-3p	down	serum	miRNA array and qRT-PCR	214
Chronic lymphocytic leukemia (CLL)	miR-155-5p	up	peripheral blood mononuclear cells (PBMCs)	an in-house-developed qRT-PCR	215
Prostate cancer	miR-424-3p	up	tissues	ISH	216
Breast cancer	miR-362-3p	up	data from TCGA	-	217
Clear cell renal cell carcinoma (ccRCC)	miR-130b, miR-18a, and miR-223	up	data from TCGA	-	218
Breast cancer	miR-196a	up	data from METABRIC	-	145

qRT-PCR: quantitative real-time polymerase chain reaction; RT-qPCR: Reverse transcription-quantitative PCR; mgmiR: methylation of genomic loci encoding microRNA; qMS-PCR: quantitative methylation-specific PCR; ISH: *in situ* hybridization; FFPE: formalin-fixed paraffin-embedded; TCGA: the Cancer Genome Atlas; METABRIC: the Molecular Taxonomy of Breast Cancer International Consortium.

**Table 2:** Clinical trials with miRNA-based therapeutics

Targeted miRNA	Developmental drug or format of miRNA therapeutics	Indication	Phase
miR-16	Mesomir	Malignant pleural mesothelioma	Clinical Trial Phase I 219
miR-34a	MRX34	melanoma	Clinical Trial Phase II 220
miR-122	Miravirsen	Hepatitis C	Clinical Trial Phase II 221
miR-34a	MRX34	NSCLC	Clinical Trial Phase I 155
miR-634	miR-634-LNP	Pancreatic Cancer	*in vitro* 151
miR-429	Pre-miR-429	Pancreatic Cancer	*in vitro* 222
miR-126	miRNA mimic	Breast cancer	*in vitro* 147
miR-151a	miRNA mimic	Glioblastoma	*in vitro* and *in vivo* 148
miR-155	Cobomarsen (MRG-106)	chronic lymphocytic leukemia	Clinical Trial Phase II 223
miR-1294	miRNA mimic	Glioma	*in vitro* and *in vivo* 224
miR-383-5p	miRNA mimic	Ovarian cancer	*in vivo* 225
miR145	miRNA145 mimics	colorectal cancer	*in vivo* 226
miR-1258	miR-1258 mimic	myeloma	*in vitro* and *in vivo* 160
miR-497	miR-497 mimic	NSCLC	*in vitro* 227

## Abbreviations

miRNAs: MicroRNAs
ncRNAs: non-coding RNAs
HUGO: Human Genome Organisation
HGNC: the HUGO Gene Nomenclature Committee
Pol II: RNA polymerase II
DGCR8: DiGeorge syndrome critical region gene 8
RanGTP: Ran guanosine triphosphate
TRBP: HIV-1 TAR RNA binding protein
Ago: Argonaute
N-domain: N-terminal domain
RISC: RNA-induced silencing complex
snoRNAs: small nucleolar RNAs
tRNAs: transfer RNAs
m7G: 7-methylguanosine
(PARN): poly(A)-specific ribonuclease
PTMs: post-translational modifications
mRNA: messenger RNA
RBPs: RNA-binding proteins
lncRNAs: long non-coding RNAs
3'-UTR: 3'-untranslated region
A: adenine
GW182: a 182 kDa glycine-tryptophan protein
DCP1-DCP2: mRNA-decapping enzyme subunit 1 (DCP1)-DCP2
PABPC: cytosolic polyadenylate-binding protein
PAN2-PAN3: poly(A)-nuclease deadenylation complex subunit 2 (PAN2)-PAN3
CCR4-NOT: carbon catabolite repressor protein 4 (CCR4)-NOT
XRN1: 5′-3′ exoribonuclease 1
DDX6: DEAD-box protein 6
eIF4A: eukaryotic translation initiation factor 4 A
MREs: miRNA-recognition elements
CDS: coding sequences
OSCC: oral squamous cell carcinoma
CDK6: cyclin-dependent kinase 6
ADAM17: A disintegrin and metalloproteinase 17
HCC: hepatocellular carcinoma
PIK3R1: phosphoinositide-3-Kinase regulatory subunit 1
CRC: colorectal cancer
EBV: Epstein-Barr virus
NSCLC: non-small cell lung cancer
BCL-2: B-cell lymphoma 2
FasL: Fas ligand
HIF-1α: Hypoxia-inducible factor 1α
VEGF: vascular endothelial growth factor
EMT: epithelial-mesenchymal transition
IFN-γ: interferon-γ
NK: natural killer
MHC: major histocompatibility complex
MICA: major histocompatibility complex class I-related proteins A
NKT: natural killer T
HLA: human leukocyte antigens
APM: class I antigen processing machinery
ICPs: immune checkpoint proteins
TILs: tumor-infiltrating lymphocytes
Th1 cells: T helper 1 cells
CTLs: Cytotoxic T-cell lymphocytes
MDSCs: myeloid-derived suppressor cells
AML: acute myeloid leukemia
TAMs: tumor-associated macrophages
IL-10: interleukin 10
CAFs: cancer-associated fibroblasts
ceRNAs: endogenous RNAs
circRNAs: circular RNAs
snRNAs: small nuclear RNAs
NPC: nasopharyngeal carcinoma
PCAT7: prostate cancer associated transcript 7
ESCC: esophageal squamous cell carcinoma
ANRIL: antisense noncoding RNA in the INK4 locus
GBC: gallbladder carcinoma
GBM: glioblastoma multiforme
eIF4A3: eukaryotic initiation factor 4A3
PPIAP43: peptidyl-prolyl cis-trans isomerase A pseudogene 43
PPIA: peptidyl-prolyl cis-trans isomerase A
SCLC: small cell lung cancer
TDMD: target RNA-directed miRNA degradation
qRT-PCR: quantitative real-time polymerase chain reaction
RT-qPCR: Reverse transcription-quantitative PCR
mgmiR: methylation of genomic loci encoding microRNA
qMS-PCR: quantitative methylation-specific PCR
FFPE: formalin-fixed paraffin-embedded
CSF: cerebrospinal fluid
PBMCs: peripheral blood mononuclear cells
TCGA: the Cancer Genome Atlas
METABRIC: the Molecular Taxonomy of Breast Cancer International Consortium
PPGLs: pheochromocytomas or paragangliomas
HNSCC: head and neck squamous cell carcinoma
FNA: fine needle aspiration
CNS: central nervous system
PCNSL: primary central nervous system lymphoma
GCTs: Germ cell tumours
SS: Synovial sarcoma
TNBC: triple-negative breast cancer
ESCC: esophageal squamous cell carcinoma
PDAC: pancreatic ductal adenocarcinoma
CLL: chronic lymphocytic leukemia
EC: endometrial cancer
ccRCC: clear cell renal cell carcinoma
DPV: differential pulse voltammetry
ex-miRNAs: exosomal serum miRNAs
ISH: *in situ* hybridization
FN1: Fibronectin 1
SNHG1: small nucleolar RNA host gene 1
Dox: doxorubicin
DSB: double-strand break
HER2: human epidermal growth factor receptor 2
CAR: chimeric antigen receptor
EGFR: epidermal growth factor receptor
Glut-1: glucose transporter 1
EGFR: epidermal growth factor receptor
PDL1: programmed death ligand 1
PAC: pancreatic adenocarcinoma
OS: overall survival
DFS: disease-free survival
nCT: neoadjuvant chemotherapy
ERα36: estrogen receptor alpha 36
ADME: absorption distribution metabolism and excretion
CYP: cytochrome P450
ABCG2: ATP-binding cassette sub-family G member 2
MDR1: multidrug resistance 1
P-gp: P-glycoprotein
STAT3: signal transducer and transcriptional activating factor 3
mTOR: mammalian target of rapamycin
RPA: replication Protein A
PTEN: phosphatase and tensin homologous protein
DSB: double-strand break
ERCC1: excision repair cross-complementing 1
